# Ewing sarcoma family of tumors-derived small extracellular vesicle proteomics identify potential clinical biomarkers

**DOI:** 10.18632/oncotarget.27678

**Published:** 2020-08-04

**Authors:** Glenson Samuel, Jennifer Crow, Jon B. Klein, Michael L. Merchant, Emily Nissen, Devin C. Koestler, Kris Laurence, Xiaobo Liang, Kathleen Neville, Vincent Staggs, Atif Ahmed, Safinur Atay, Andrew K. Godwin

**Affiliations:** ^1^Division of Pediatric Hematology Oncology and Bone Marrow Transplantation, Children’s Mercy Hospital, Kansas City, MO, USA; ^2^Department of Pediatrics, University of Missouri-Kansas City, Kansas City, MO, USA; ^3^University of Kansas Cancer Center, Kansas City, KS, USA; ^4^Department of Pathology and Laboratory Medicine, University of Kansas Medical Center, Kansas City, KS, USA; ^5^Clinical Proteomics Laboratory, Department of Medicine, University of Louisville, Louisville, KY, USA; ^6^Robley Rex VA Medical Center, Louisville, KY, USA; ^7^Department of Biostatistics & Data Science, University of Kansas Medical Center, Kansas City, KS, USA; ^8^Department of Pediatrics, University of Arkansas for Medical Sciences, Little Rock, AR, USA; ^9^Biostatistics & Epidemiology Core, Children’s Mercy Hospital, Kansas City, MO, USA; ^10^Department of Pathology, Children’s Mercy Hospital, Kansas City, MO, USA; ^11^Bristol-Myers Squibb, Cambridge, MA, USA; ^*^These authors contributed equally to this work

**Keywords:** Ewing sarcoma, EWS-ETS, biomarkers, extracellular vesicles, exosomes

## Abstract

Purpose: Ewing Sarcoma Family of Tumors (ESFT), the second most common pediatric osseous malignancy, are characterized by the pathognomonic chromosomal *EWS-ETS* translocation. Outside of tumor biopsy, no clinically relevant ESFT biomarkers exist. Additionally, tumor burden assessment at diagnosis, monitoring of disease responsiveness to therapy, and detection of disease recurrence are limited to radiographic imaging. To identify new, clinically relevant biomarkers we evaluated the proteome of a subset of ESFT-derived small extracellular vesicles (sEVs).

Materials and Methods: We performed the first high quality proteomic study of ESFT-derived sEVs from 5 ESFT cell lines representing the most common *EWS-ETS* fusion types and identified 619 proteins composing the core ESFT sEV proteome. We compared these core proteins to databases of common plasma-based proteins and sEV-associated proteins found within healthy plasma to identify proteins unique or enriched within ESFT.

Results: From these analyses, two membrane bound proteins with biomarker potential were selected, CD99/MIC2 and NGFR, to develop a liquid-based assay enriching of ESFT-associated sEVs and detection of sEV mRNA cargo (*i.e., EWS-ETS* transcripts). We employed this immuno-enrichment approach to diagnosis of ESFT utilizing plasma (250 μl) from both localized and metastatic ESFT pediatric patients and cancer-free controls, and showed significant diagnostic power [AUC = 0.92, *p* = 0.001 for sEV numeration, with a PPV = 1.00, 95% CI = (0.63, 1.00) and a NPV = 0.67, 95% CI = (0.30, 0.93)].

Conclusions: In this study, we demonstrate utilization of circulating ESFT-associated sEVs in pediatric patients as a source of minimally invasive diagnostic and potentially prognostic biomarkers.

## INTRODUCTION

Ewing Sarcoma Family of Tumors (ESFT) encompass a group of highly aggressive pediatric osseous and soft tissue malignancies thought to originate from primordial bone marrow-derived mesenchymal stem cells and consists of small round blue cells with minimal stroma and differentiation [1]. Ewing sarcoma of the bone, extraosseous Ewing sarcoma, and peripheral primitive neuroectodermal tumors (pPNET) are all considered manifestations of a single neoplastic entity. With a peak incidence within the second decade of life, slight male preponderance, high incidence in those of European ancestry and approximately 3 cases/million/year [2], this malignancy continues to remain the second most prevalent pediatric bone tumor after osteosarcoma. ESFT can occur in any bone; however, most typical sites of involvement are the pelvis (25%), femur (16%), ribs (13%), spine (8%), and scapula (5%) [3]. Overt metastatic disease is prognostic, with approximately 25% of newly diagnosed ESFT being affected [4]. Of the ESFT metastatic patients, 37% (or 9% of all ESFT patients) have metastases confined to the lung or pleura [5].

Overt metastatic disease is prognostic; however, it is evident that the preponderance of ESFT patients (even with localized disease) harbor micrometastatic disease. Hence, the necessity for a multidisciplinary approach in the management of these patients, involving interval compression chemotherapy with that of local control (surgical and/or radiation) as well. Localized ESFT patient outcomes based on the most recent Children’s Oncology Group completed trial (AEWS0031) is 73% Event Free Survival (EFS) at 5 years [6]. Outcomes continue to remain dismal for pediatric metastatic ESFT, with a 20–30% 2- to 3-year EFS. Despite the intensification of therapies (interval-compressed VDC/IE) and improved local control in pediatric ESFT, 30–40% of patients experience recurrence [7]. Most recurrences occur within 2 years from time of diagnosis, with an EFS of less than 10% at 3 years for early recurrence (< 2 years from diagnosis) [8] and for late recurrences (> 2 years from diagnosis) greater than 25% OS (overall survival). The median relapse-free interval (time of diagnosis to first recurrence) is 17 months (range 5–90 months) [5]. Outside of the presence of overt metastatic disease, no clinically relevant predictive biomarkers exist which are indicative of the increased risk of recurrence in localized ESFT patients. Various other clinical prognostic factors such as age (> 15 years of age with worse outcomes), tumor location (pelvic tumors with worse outcomes) [6], tumor size (larger tumors > 100 mL with worse outcomes) [9] and histologic response to induction therapy (poor response with worse outcomes) [10]; however, have not been incorporated within pediatric ESFT treatment risk stratification [11]. Standard circulating tumor markers are not applicable to ESFT. Serum lactate dehydrogenase (LDH) has been most consistently associated with aggressive disease, but lacks ESFT specificity [12].

The clinical presentation of ESFT are often non-specific in nature, with pain, swelling and discomfort consisting of the most typical complaints and are all related to growth of these tumors. According to the literature, there typically is a lapse of three to nine month range from onset of symptoms prior to time of initial diagnosis, thus delaying initiation of oncological management [5]. Currently, diagnostic and monitoring modalities for children and young adults with ESFT require utilization of radiographic imaging and the important diagnostic testing via biopsies of suspicious lesions for establishment of diagnosis and prognosis in these patients. Although the standard of care, these tests are expensive, invasive and associated with potential long-term risks. Traditionally, ESFT was considered a diagnosis of exclusion, but over the past few decades, with the introduction of immuno-histochemical markers, *e.g.,* CD99/MIC2 and detection of the oncogenic chimeric fusion involving the Ewing sarcoma RNA (ribonucleic acid) binding protein 1 gene (*EWSR1* gene; Ewing sarcoma breakpoint region 1) [13], which is a hallmark of ESFT, the accuracy of diagnosis has considerably improved. However, these approaches require invasive open or core biopsy sampling of active tumor tissue [14]. The most utilized immunohistologic stain in ESFT diagnosis is the monoclonal antibody CD99 (MIC-2), which recognizes the cell surface protein. ESFT specimens demonstrate a crisp and strong membranous positivity with CD99 antibody in more than 90% to 95% of cases reported. Therapeutic response assessment is based upon tumor size changes as determined with anatomic imaging tests. Utilization of FDG PET-CT in staging, restaging and assessment of response to ESFT therapy is increasing worldwide although not considered a standard in the diagnostic workup [15]. Even children and young adults successfully treated for their localized ESFT, are at high risk of relapse, and must be monitored for years by periodical medical imaging examinations, often resulting in additional X-ray exposure. Absence of asymptomatic ESFT diagnostic biomarkers has lent to the reliance on clinical symptomatology and/or findings with complementary conventional imaging modalities including FDG PET-CT, to detect and monitor these patients. However, imaging in and of itself is a poor means for early cancer detection and monitoring of recurrence. Therefore, the discovery of new ESFT biomarkers and development of clinically useful tests for early detection and monitoring disease progression are considerably in need.

There has been a momentum towards the direction of personalized medicine, especially in solid pediatric tumors such as ESFT and other pediatric sarcomas [16]. It naturally follows that the identification of novel and robust biomarkers as well as the tools to effectively measure them are in dire need. Many of the biomarkers studied regarding ESFT have been prognostic in nature and rely upon biopsy/resection of tumor tissue [17, 18]. Currently, there are no readily available clinical liquid-based assays utilizing biological fluids such as blood, serum, or urine specifically for diagnosing ESFT, evaluating minimal residual disease, or monitoring of disease progression [4].

To address some of these diagnostic hurdles, we turned our attention to a class of circulating extracellular vesicles (EVs), of which small EVs (sEVs) or exosomes have gained considerable traction in the field of liquid-based biomarkers. sEVs/exosomes are proving to be an abundant source of protein- and nucleic acid-related biomarkers [19–22]. Exosomes originate through the formation of multivesicular bodies (MVB) within the endosomal compartment of cells [23] and are secreted into the extracellular space as a result of fusion with the cellular plasma membrane. sEVs contain a varying assortment of proteins, lipids, and nucleic acids reflective of their cell of origin. The population of sEVs within the blood is heterogenous because circulating extracellular vesicles are released by most if not all types of cells in the body. It is estimated that exosomes released by platelets, lymphocytes, dendritic cells, and other immune cells comprise 80–90% of serum/plasma exosomes [24]. In contrast to other classes of extracellular vesicles, tumor derived sEVs/exosomes, have been found to be elevated within the circulation of cancer patients and reflective of their tumor burden [25, 26]. The cell specific cargo of sEVs, including a wide array of proteins and RNAs (*e.g.,* mRNA, miRNA, and LncRNA), has been shown to have biomarker potential in several malignancies including; gastrointestinal stromal tumor [25], pancreatic cancer [27], acute myeloid leukemia [28] and glioblastoma multiforme [29]. In addition, our group and others, have shown that tumor derived sEVs play pivotal roles in intercellular communication, tumor development, angiogenesis [30], preparation of pre-metastatic niches [31], modulating anti-tumor immune responses [32] and drug resistance [33]. Tumor derived sEVs are found abundantly circulating in blood plasma and within malignant effusions derived from cancer patients [34, 35]. Furthermore, previous work has suggested that tumor derived sEVs may provide a biomarker source for ESFT [36]. The essential role of *EWS-ETS* fusion transcripts makes it less likely to be down-regulated or non-existent during tumor progression, convincingly supporting their routine utility for diagnostic assessment in ESFT tumor biopsied tissues but also in circulating tumor derived sEVs. In this study we report for the first time the proteome of ESFT-derived sEVs/exosomes and develop a clinically useful test based on immuno-enrichment of ESFT-sEVs and detection of the *EWS-ETS* fusion transcripts.

## RESULTS

### Ewing sarcoma family of tumor cell lines constitutively release EWS-ETS transcript and oncoprotein in association with sEVs

**Figure 1:**
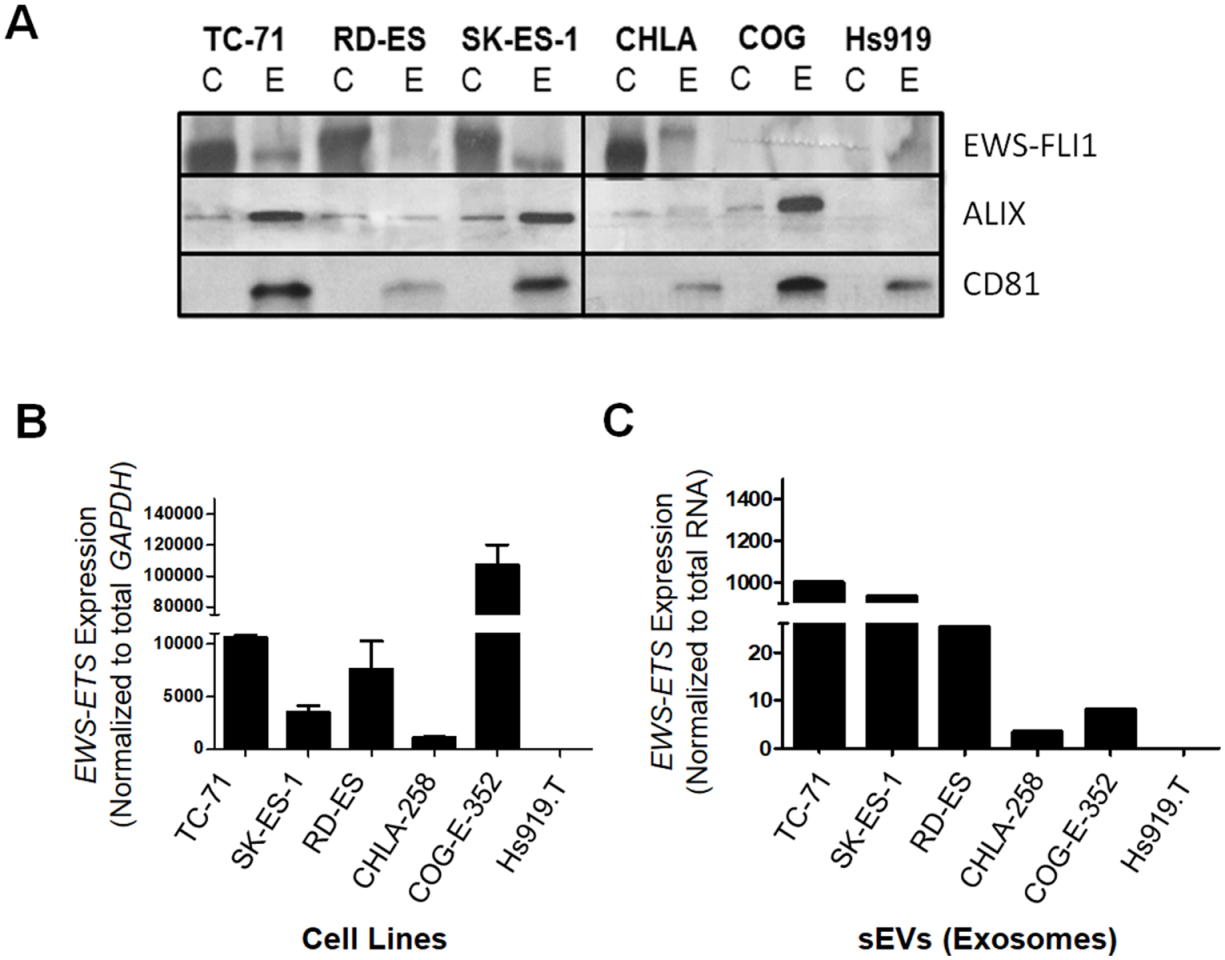
*EWS-ETS* transcript and protein are detected in ESFT cell lines and their corresponding sEVs. (**A**) Western blot analysis of EWS-FLI1 protein and common exosomal markers (ALIX, CD81) in ESFT cell lines (C) and sEVs (E) from TC-71, RD-ES, SK-ES-1, CHLA-258, and COG-E-352 and Hs919.T, a benign osteoid osteoma cell line, was used as a negative control. EWS-FLI1 is detected using an antibody directed at the C-terminal region of FLI1. ALIX and CD81 common markers of exosomal and small EV populations. (**B**) Expression of the *EWS-FLI1* or *EWS-ERG* fusion transcripts in ESFT and Hs919.T (negative control) cell lines. Expression values were normalized to the housekeeping gene *GAPDH*. (**C**) Expression levels of *EWS-FLI1* or *EWS-ERG* fusion transcripts in ESFT cell line derived sEVs normalized to total RNA input.

For this study, ESFT cell lines representative of the most common *EWS-ETS* fusion types; namely *EWS-FLI1* type I (TC-71), *EWS-FLI1* type II (RD-ES and SK-ES-1), *EWS-FLI1* type III (CHLA-258) fusions, as well as COG-E-352 which carries the *EWS-ERG* fusion were used as sources for sEVs. In addition to these ESFT cell lines, Hs919. T, a benign osteoid osteoma cell line known to lack *EWS-ETS* fusions was included as a negative control (Supplementary Table 1) [37]. sEVs from each of the ESFT and control cell lines were isolated using ultracentrifugation [38] and the contents of the resulting 120,000 × *g* pellet were then characterized for markers and size distribution (Figure 1 and Supplementary Figure 1). Nanoparticle Tracking Analysis (NTA) indicated that all five ESFT cell lines released a homogenous mixture of nano-sized vesicles with varying diameters between 150–239 nm (Supplementary Figure 1), consistent with previous reports for sEVs [39]. In addition, western blot analysis confirmed an enrichment of exosomal markers, Alix and CD81, in sEVs as compared to the parental cells-derived lysates (Figure 1A), further supporting the purity of the isolated sEVs. Using a monoclonal antibody raised against the C-terminal domain of FLI1, the expression of the EWS-FLI1 fusion protein was also only detectable in sEVs derived from cell lines carrying the *EWS-FLI1* fusion transcripts (Figure 1A), while COG-E-352 (*EWS-ERG)* and Hs919. T cell line derived sEVs were negative. Of note, size differences in EWS-ETS oncoproteins were noticeable between cellular extracts and their corresponding sEV lysates, suggesting these fusion proteins and other sEV proteins may be post-translationally modified prior to sorting into sEVs as previously been described [40]. Next RT-PCR analysis demonstrated and validated that ESFT cell lines known to have an *EWS-ETS* fusion were positive for either the *EWS-FLI1* or *EWS-ERG* transcripts (Figure 1B). Most importantly, enrichment of the *EWS-ETS* transcripts were detectable within each of the ESFT cell line derived sEVs while absent within Hs919. T cells-derived sEVs (Figure 1C). In addition to PCR we evaluated the mRNA content of both cell lysates and their corresponding sEVs using a portion of the Nanostring Elements sarcoma panel as previously published [41]. While we were able to detect and differentiate *EWS-ETS* fusion transcripts in cellular mRNA, we were unable to detect the presence of the fusion transcript within sEV samples using the nCounter platform (Nanostring) and a custom designed EWS-ETS fusion transcript panel (data not shown), which led us to choose qPCR as our primary assay for transcript detection. Taken together these data demonstrate that sEVs released from ESFT cell lines harbor the pathognomonic EWS-ETS fusion protein and transcript characteristic of ESFT.

### Proteomic analysis of ESFT cell lined derived sEVs identify a core set of ESFT-associated exo-proteins

Two biological replicates of ESFT sEV preparations were isolated from TC-71 and CHLA-258, while one was isolated from RD-ES, SK-ES-1, and COG-E-352 cell lines derived conditioned media. Each biological replicate was analyzed as technical replicates to further establish a reliable sEV proteome (*i.e.,* exo-proteome) profile.

**Figure 2:**
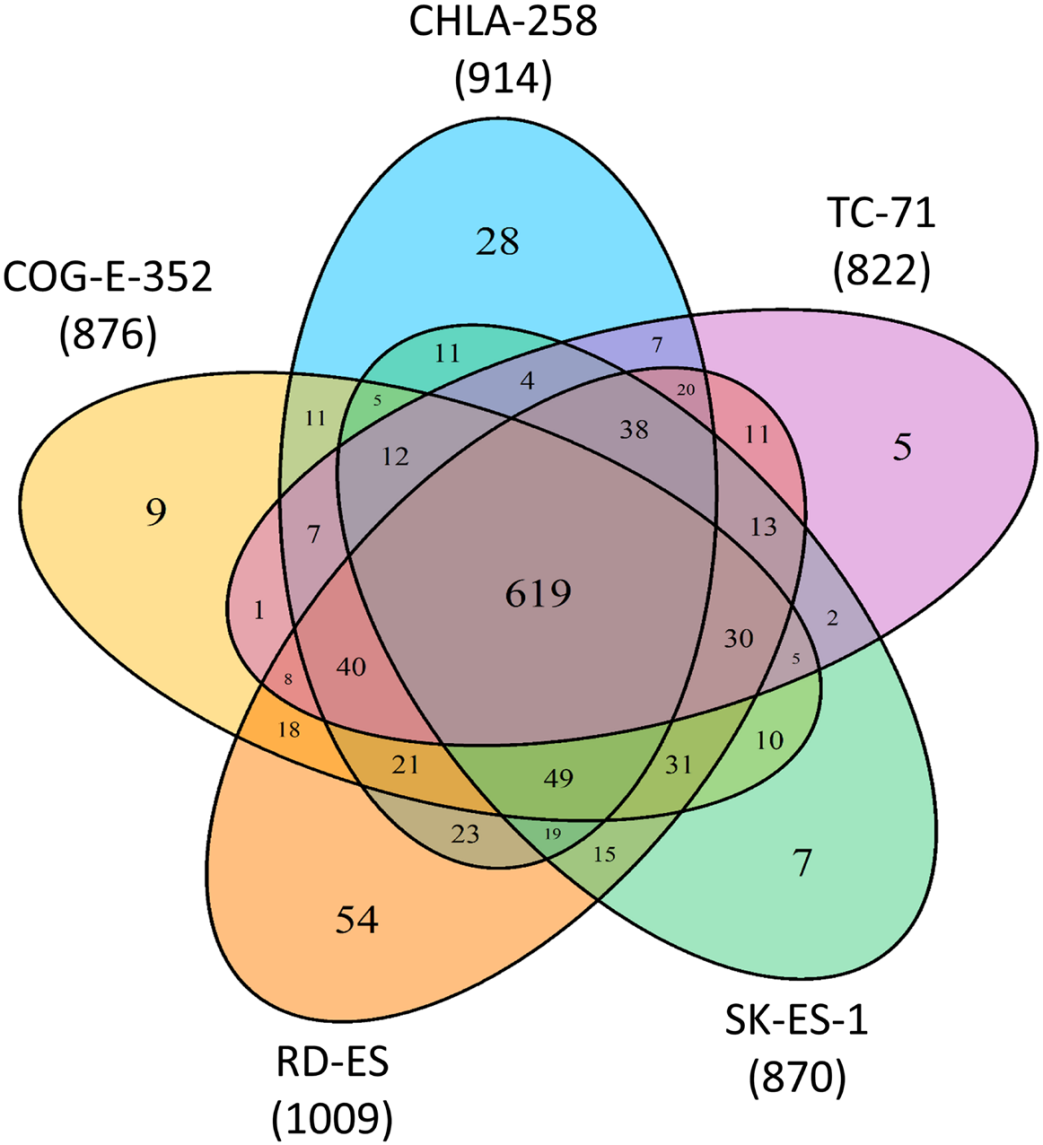
Common and unique proteins to ESFT cell line derived sEVs. Venn diagram depicting quantitative measurements from spectrometry analysis of 5 Ewing Sarcoma Family of Tumors (ESFT) cell line (EWS-FLI1 fusion Type I, II, III, and EWS-ERG fusion) derived sEVs.

Our initial analysis of ESFT cell line derived sEV proteomic data utilizing the Proteome Discoverer v1.3.0.330 (Supplementary Table 2) we determined the qualitative presence and distribution of identified proteins (total 1,082 proteins) between the five pediatric ESFT cell line derived sEVs, revealing a common subset of 619 sEV proteins out of 822 (TC-71), 870 (SK-ES-1), 876 (COG-E-352), 914 (CHLA-258), and 1,009 (RD-ES) unique peptides, representing an 75%, 71%, 71%, 68%, and 61% overlap, respectively (Figure 2). This observation was further strengthened, *i.e.,* of the total proteins (1,082) identified between the five cell lines, only 5 proteins (0.5%) were enriched solely in TC-71, 7 (0.6%) in SK-ES-1, 9 (0.8%) in COG-E-352, 28 (2.6%) in CHLA-258, and 54 (5%) in RD-ES sEVs (Figure 2).

**Figure 3:**
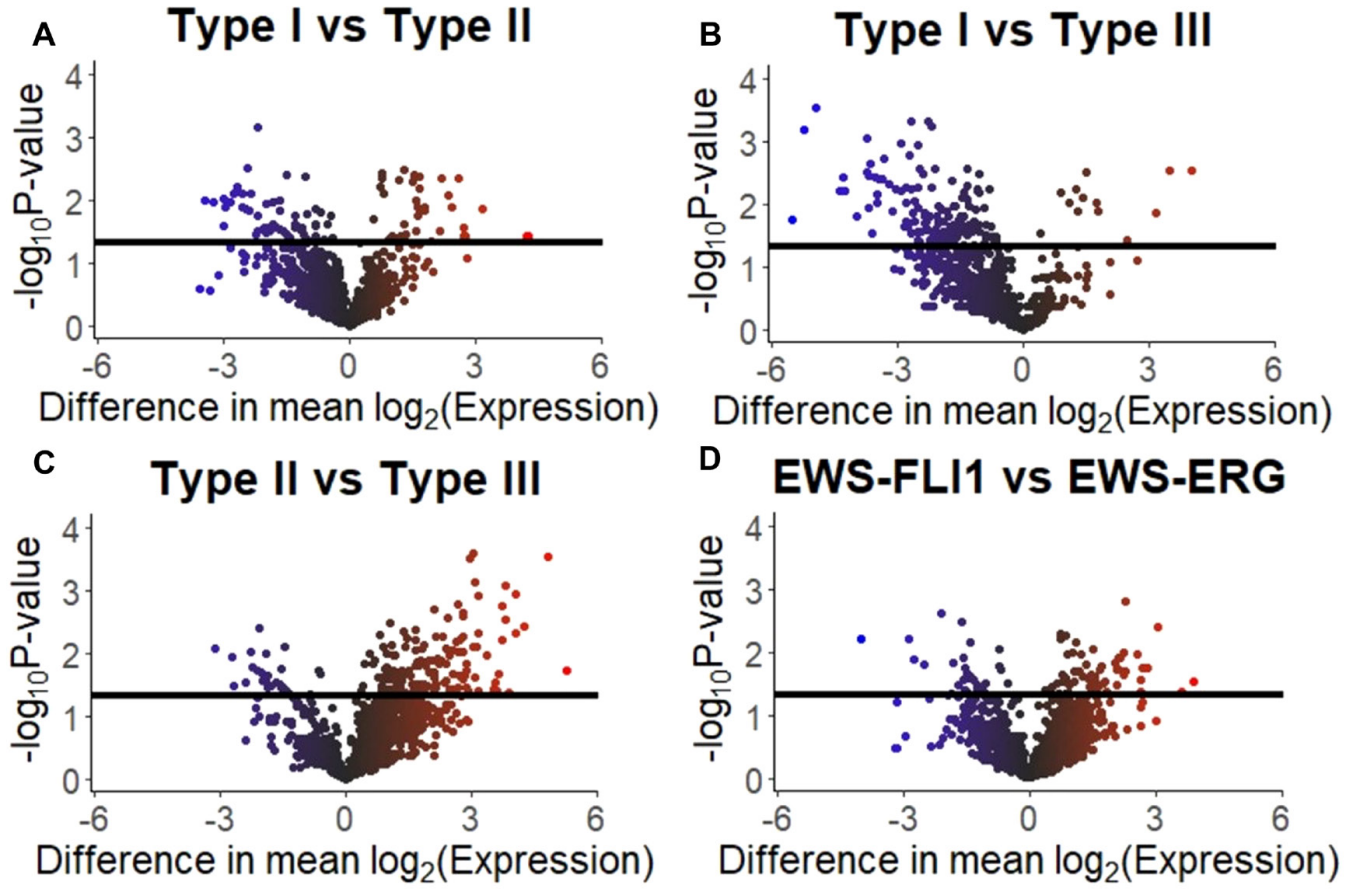
Differentially expressed proteins in ESFT cell line derived sEVs. Volcano plots visualizing the results of the protein expression analysis based on the four comparisons of interest: (**A**) Type I versus Type II fusions, (**B**) Type I versus Type III fusions, (**C**) Type II versus Type III fusions, and (**D**) EWS-FLI1 versus EWS-ERG. Horizonal black bars represent a *p*-value of 0.05 [e.g., –log10(0.05)]. The color of the plotted protein represents the difference in mean expression. The scale is –6 to 0 to 6 with the color scale as blue to black to red, respectively.

Analysis of the mass spectrometry proteomic data from sEVs derived from ESFT cell lines, EWS-FLI1 Type I (TC-71, *n* = 2), EWS-FLI1 Type II (SK-ES-1, *n* = 1 and RD-ES, *n* = 1), EWS-FLI1 Type III (CHLA-258, *n* = 2) and EWS-ERG (COG-E-352, *n* = 1) were analyzed utilizing the total averaged spectrum counts (Supplementary Table 3) for differential protein expression comparison analysis. For EWS-FLI1 Type I versus Type II a differential expression in 437 proteins was noted, while 322 proteins in EWS-FLI1 Type I vs Type III, 241 proteins in EWS-FLI1 Type II vs Type III, and 572 for EWS-FLI1 vs EWS-ERG. (Supplementary Table 4 and Figure 3).

### Pathway analysis of proteomic data

Mass spectrometry proteomic data via the Proteome Discoverer v1.3.0.330 was utilized to gain an understanding of the origin of the core ESFT sEV proteome using the Functional Enrichment analysis tool (FunRich). Unsurprisingly, the largest cellular component of origin was listed as ‘extracellular exosomal’ (~80%) (Supplementary Figure 2A). Many of the shared ESFT sEV proteins were found to be ubiquitously exosomal proteins, such as membrane transport and fusion proteins (Annexins A1, A11, A2, A5, A6, A7), tetraspanin protein CD81, chaperone proteins (heat shock family protein 70, HSP70 and heat shock protein family 90, HSP90), metabolic enzymes (pyruvate kinase, ATPase, G6P-isomerase, glyceraldehyde 3-phosphate dehydrogenase, enolase, aldolase, phosphoglycerate kinase 1), antigen presenting proteins (MHC-1, H2A and complement), and cytoskeletal structural proteins (ARP2, ARP3, cofilin 1, moesin, actin gamma 1, syndecan binding protein) (Supplementary Figure 2).

To gain a further understanding of the functional classification of ESFT sEV proteins, we used Protein ANalysis THrough Evolutionary Relationships [42] 10.0 Gene ontology Molecular Function software. This software allowed the identification of top molecular functions, biological processes, and protein classes within our ESFT sEV proteome. We identified 288 different molecular functions with the uppermost consisting of protein binding, catalytic activity, and structural molecule activity (Supplementary Figure 2B). Out of 550 identified cellular processes, proteins involved within the metabolome, cellular component organization and biogenesis, biological regulation, localization processes, and response to stimulus were most enriched in ESFT derived sEVs (Supplementary Figure 2C). The bulk of protein class consisted of nucleic acid binding, hydrolase, enzyme modulator, cytoskeletal protein, and chaperone proteins (Supplementary Figure 2D). This analysis demonstrates that ESFT derived sEVs carry a wide variety of proteomic content composed of both common sEVs elements and others which may be more specifically characteristic of ESFT.

To identify disrupted biological pathways between EWS-FLI1 versus EWS-ERG cell line derived sEVs, a gene set enrichment analysis (GSEA) was implemented. This analysis demonstrated two statistically significant pathways enriched between the comparison groups, the ECM-receptor interaction (*p*-value: 0.012) and the focal adhesion pathway (*p*-value: 0.026) (Supplementary Table 5).

### Identification of candidate ESFT sEVs proteins as potential biomarkers

Subsequent to this proteomic analysis, we then asked whether any of these ESFT sEV-proteins could serve as potential ESFT biomarkers and be exploited to specifically enrich for circulating tumor-derived sEVs. The human plasma constitutes of approximately 7% proteins and considered the most complex human-derived proteome containing other tissue proteomes as subsets [43]. To reach the full potential of ESFT derived sEVs as a source of biomarkers and distinguish their proteome from contaminating plasma proteins, it is essential to differentiate specific ESFT derived sEV proteins applying a large robust normal/healthy proteome dataset. The Plasma Proteome Project (http://www.plasmaproteomedatabase.org/) is the characterization of the human plasma proteome by an international consortium and is one of the largest resources of proteomics reported. All proteins previously reported to be found within the plasma and serum of healthy individuals were eliminated by manually comparing our initial proteomic ESFT cell line derived sEV dataset (Proteome Discoverer v1.3.0.330 based data set) to that of the proteins in the Plasma Proteome Database. This approach identified a total of 60 potential ESFT biomarker candidates. Even though present in ESFT-derived sEVs, CD99/MIC2 and HINT1 were overlooked based on this type of analysis. However, given their documented importance related to ESFT (discussed below) we reincorporated them onto the candidate list resulting in a total of 62 protein biomarkers (Supplementary Table 6).

**Figure 4:**
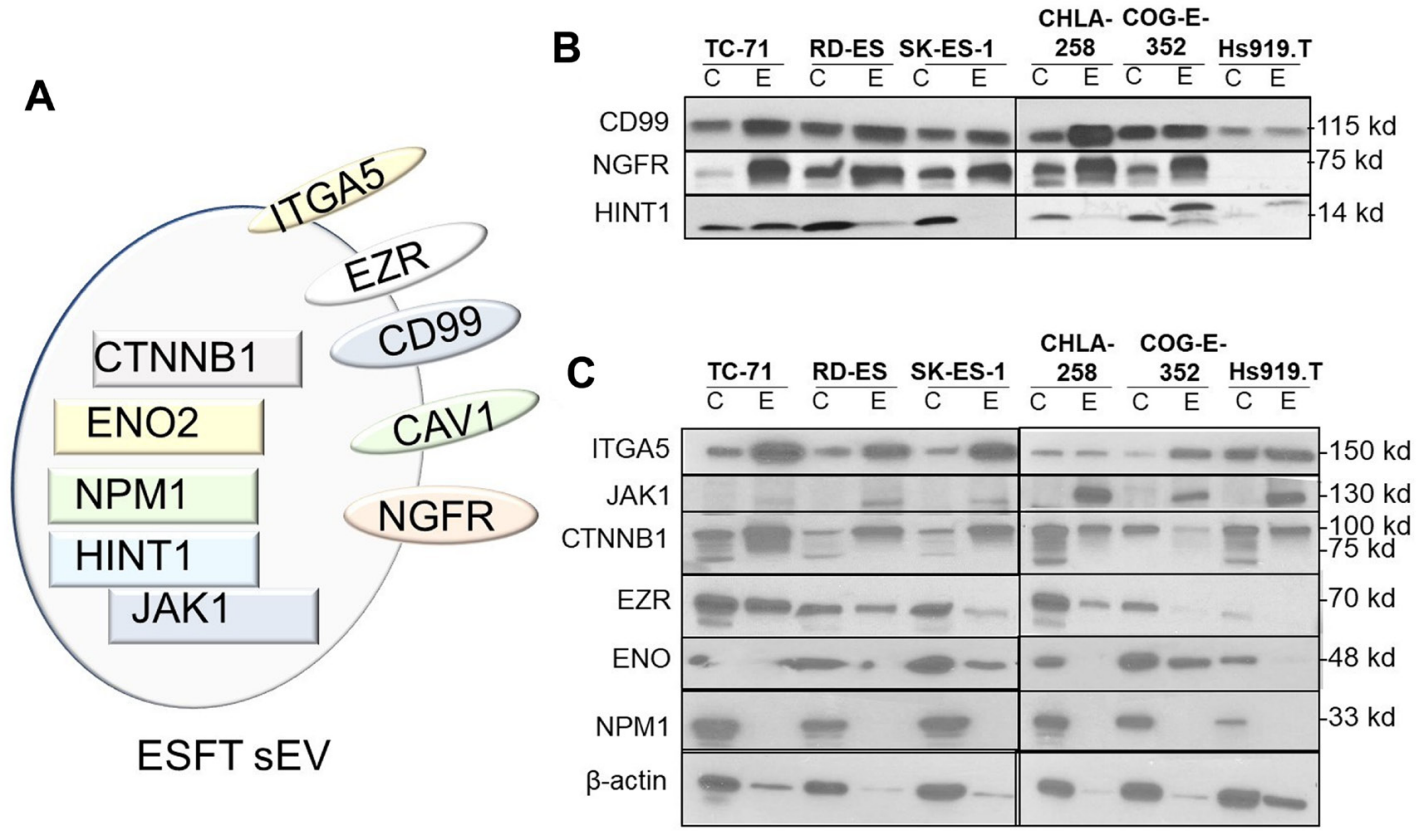
Identification of potential sEV-associated ESFT biomarkers. (**A**) Cellular localization of potential ESFT sEV biomarkers (Intraluminal, rectangles; plasma membrane, ovals). (**B**–**C**) Validation of ITGA5 (Integrin Subunit Alpha 5), JAK1 (Janus Kinase 1), CTNNB1 (Catenin Beta 1), EZR (Ezrin), ENO (Enolase), and NPMN1 (Nucleophosmin 1) as potential sEV-protein biomarkers for ESFT by western blot analysis.

We then utilized the Ingenuity Pathway Analysis (IPA) software platform for gene ontology and pathway analysis to elucidate biomarkers previously published within sarcomas (QIAGEN Inc., https://www.qiagenbioinformatics.com/products/ingenuitypathway-analysis). IPA is a curated database that utilizes current knowledge available on genes, proteins, normal cellular and pathological processes, signaling and metabolic pathways, required for pathway construction. We utilized the Ingenuity Biomarker Analyzer tool, to identify cellular biological function and canonical pathways in known proteins previously identified as biomarkers. Present within this data set were immuno-histochemical markers commonly expressed in ESFT, *e.g.,* CD99/MIC2 and CAV1 and are used clinically to differentiate ESFTs from other small round cell tumors. Another marker identified was Histidine triad nucleotide binding protein 1 (HINT1). HINT1 has been shown to repress β-catenin-mediated transcription of Wnt target genes and had been noted to be differentially expressed between localized and metastatic ESFT [44]. All together a total of 10 proteins previously identified to be associated with sarcomas were further investigated (Supplementary Table 7 and Figure 4A).

Our next step was to then confirm the presence and enrichment of these proteins within our cell lines and cell line-derived sEVs by western blot analysis (Figure 4B and 4C). This analysis corroborated that CD99 and NGFR were expressed within ESFT cell lines and enriched within ESFT-derived sEV. The protein levels were significantly lower or absent in the control Hs919. T cells and its associated sEVs (Figure 4B). Likewise, HINT1, EZR, and ENO were enriched in a majority of ESFT sEV samples and minimally enriched or absent in control (Figure 4B and 4C). ITGA5 (Integrin Subunit Alpha 5), JAK1 (Janus Kinase 1), NPM1 (Nucleophosmin), and CTNNB1 (Catenin Beta 1) were either not detected in ESFT or not substantially enriched in ESFT samples as compared to the control (Figure 4C). Taken together, this analysis lead towards the identification of 5 proteins, (2 membrane bound and 3 cytosolic) with the potential as ESFT sEV biomarker capability. We next evaluated the two most promising membrane-associated biomarkers (NGFR and CD99/MIC2) by IHC on primary tumor biopsies. Over 90% of tumor biopsies expressed high levels for membranous CD99. For NGFR, over 50% of tumors stained between medium and high levels and less than 18% were negative for the marker (Supplementary Figure 3).

### Immunoprecipitation with NGFR and CD99 enriches for tumor derived sEVs

**Figure 5:**
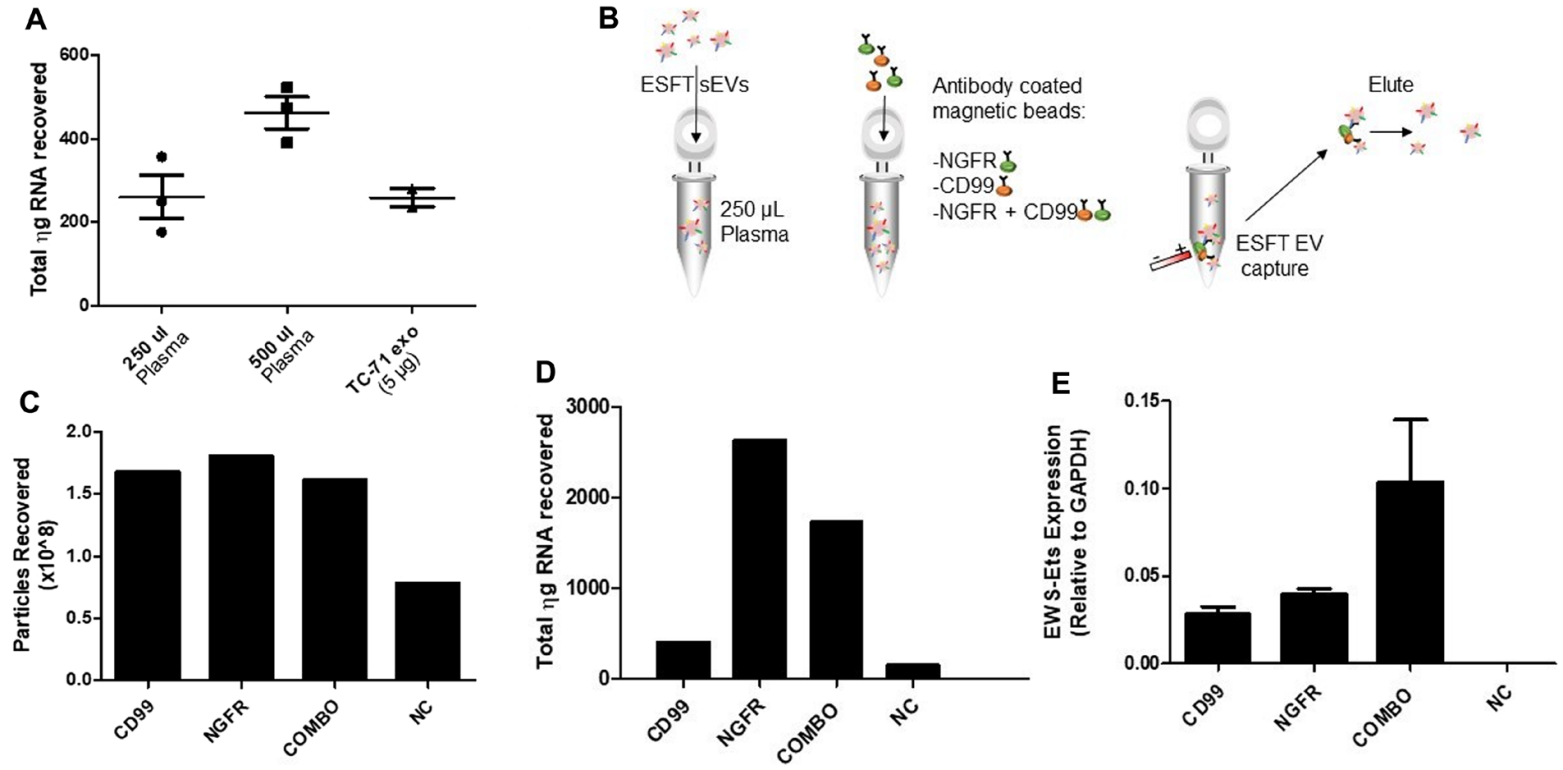
Development of an IP to enrich for ESFT-specific sEVs. (**A**) Amount of total RNA (in nanograms) recovered from sEVs isolated through ultracentrifugation from human plasma samples (250 μL or 500 μL) and from TC-71 derived sEVs as a control. (**B**) Schematic depicting the methodology of our IP strategy. (**C**–**E**) Particles recovered (C), total RNA (in ng) recovered (D), and the relative abundance of *EWS-ETS* transcripts (E) obtained from sEVs isolated from sEV-spiked plasma samples. EWS-ETS expression comparison utilizing CD99 versus NGFR versus CD99 + NGFR (COMBO) antibody cocktail for enrichment of ESFT sEVs. The COMBO demonstrated greater yield for increased enrichment of ESFT sEVs.

To begin to develop a clinical assay based on circulating tumor derived sEVs, we opted to exploit the membranous location of CD99 and NGFR to develop a tool capable of enriching ESFT-associated sEVs from plasma in pediatric patients. Our group as well as others have attempted to detect exosomal *EWS-ETS* transcripts directly from plasma, but have been relatively unsuccessful. To begin, we determined the minimal plasma volume required for sEV detection in order to best minimize the risks of hemodynamic instability or iatrogenic anemia within our pediatric patient population. We isolated sEVs from 250 μL and 500 μL of plasma from 3 healthy individuals using ultracentrifugation (120,000 × g pellet) and then isolated total RNA from the resulting pellet. The amount of total RNA was compared to RNA isolated from a control volume (5 μg) of ESFT cell-derived sEVs (Figure 5A). From this we determined that 250 μl would be a sufficient volume for sEV RNA isolation and subsequent evaluation of *EWS-ETS* fusion transcripts. Next, we utilized healthy control plasma samples spiked with ESFT cell line derived sEVs (Figure 5B). 1 mL of plasma from healthy control samples was spiked with an excess of TC-71 derived sEVs (1 × 10^10^). From this we immunocaptured (IP) EVs from 250 μL aliquots using CD99 or NGFR antibodies alone or in 50/50 combination (combo) and with IgG as a negative control (NC) (Figure 5B). We observed little to no difference in quantification of EVs enriched from plasma with either the single antibodies or combination (combo) (Figure 5C). However, under these conditions we observed substantial non-specific binding of sEVs to the beads with IgG alone despite multiple attempts at different blocking techniques. Even given this hurdle, we obtained 1–3 μg of total RNA from sEVs isolated using either individual antibody or combo (Figure 5D). Real-time qPCR revealed that IPs with CD99, NGFR, and combo captured EVs harboring the *EWS-ETS* fusion transcript (Figure 5E). Given these data, and to ensure that we were optimizing our ESFT sEV enrichment, we opted to utilize the combo IP technique for subsequent studies. To note: EVs will be used in reference towards all particles isolated via IP method due to the IP technique itself is incapable of enriching only sEV populations.

### Identification of EWS-ETS transcripts from clinical samples

**Figure 6:**
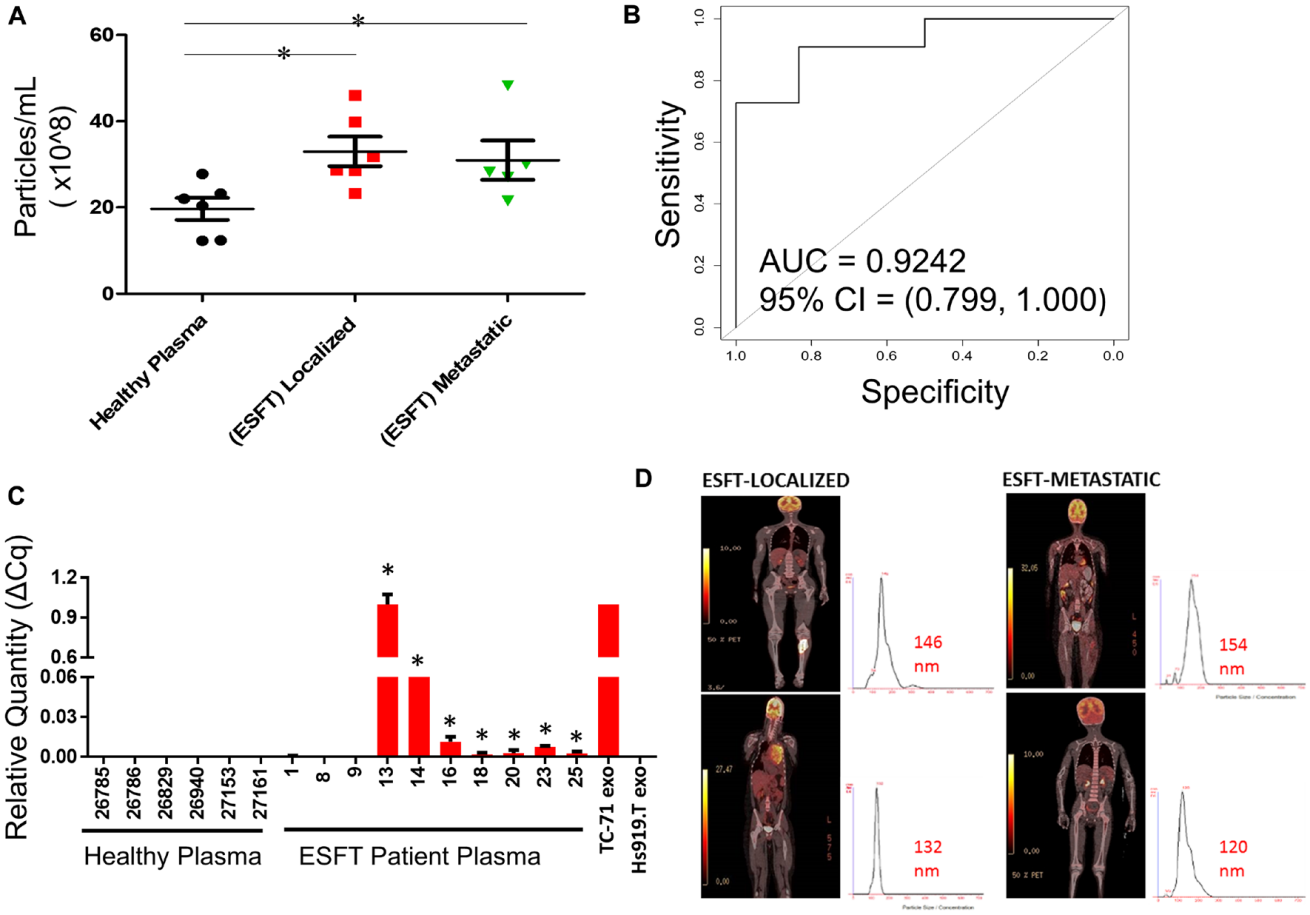
Enrichment of ESFT-sEVs from clinical ESFT plasma samples. (**A**) Total concentration of sEV’s isolated from 250 μL of pediatric ESFT (*n* = 10) and healthy plasma (*n* = 6). (**B**) Area Under the Curve (AUC) analysis (A) of total sEV counts in cases and controls following immuno-enrichment. (**C**) Detection of the *EWS-ETS* transcript by qRT-PCR, ^*^ denotes samples that crossed threshold and are considered positive, total of 7/10. cDNA from sEVs derived from TC-71 and Hs919.T cell lines served as positive and negative controls, respectively. (**D**) Localized and metastatic pediatric ESFT patient isolated sEVs demonstrating size threshold between 120–154 nm.

To transition our assay into a pre-clinical application, we isolated EVs using immuno-enrichment from 10 clinical pediatric ESFT plasma samples (ages 1 to 17 years of age) and 6 plasma samples from healthy control individuals (< 20 years of age) (Table 1). From 250 μL of healthy patient plasma we immunoprecipitated an average of 2 × 10^8^ EVs. In comparison, we isolated ~16-fold more EVs (average 32 × 10^8^ EVs, *P* = 0.01) utilizing the equivalent volume of ESFT pediatric patient plasma (Figure 6A). This was true for both subsets of ESFT patients, localized (*P* = 0.01) and metastatic (*P* = 0.04) disease. However, there was no significant variance between the number of EVs in ESFT patients with localized *vs.* metastatic disease. ROC/AUC analysis of these data resulted in a 95% confidence interval with an AUC of 0.9242 (Figure 6B). Using qRT-PCR analysis of the nucleic acid content of the isolated EVs by IP, we identified the *EWS-ETS* fusion transcript in 70% (7 of 10) of pediatric clinical samples (60% ESFT metastatic and 83% ESFT localized identified) (Figure 6C), with no false positives. cDNA from EVs derived from TC-71 and Hs919. T cell lines were used for positive and negative controls, respectively. These results equated to a positive predictive value (PPV) of 1.00 (0.63, 1.00) and negative predictive value (NPV) of 0.67 (0.30, 0.93) for detecting the *EWS-ETS* fusion transcript. To evaluate if this methodology is more efficacious than evaluating EVs as a whole, we prepared matched samples from 4 ESFT pediatric patients using immuno-isolation with combo (CD99+NGFR) or a member of the tetraspanin superfamily CD9 (a common sEV marker) in 250 μL of plasma. While no significant differences in the number of EVs were isolated (Supplementary Figure 4A), we were able to identify the *EWS-ETS* transcript in all of the ESFT patients using the COMBO immuno-isolation approach, but only 50% of the patients using CD9 antibody alone (Supplementary Figure 4B). These pre-clinical data are strongly supportive of the approach to use CD99 and NGFR for the enrichment for ESFT specific EVs from clinical patient plasma and that the proposed liquid-base biopsy can serve as a clinical tool for the diagnosis, monitoring of disease, and early detection of relapse (Figure 6D).

**Table 1:** Patient information

ID#	Age (Years)	Gender	EWSR FISH	Localized/Metastatic
1	16	M	+	Metastatic
8	6	F	+	Localized
9	9	F	+	Metastatic
13	1.6	F	+	Localized
14	6	M	+	Metastatic
16	12	F	+	Localized
18	15	F	+	Localized
20	6	F	+	Metastatic
23	16	F	+	Metastatic
25	17	M	+	Localized
026785	3	M	NA	NA
236786	20	M	NA	NA
026839	2	F	NA	NA
026940	11	F	NA	NA
027153	20	M	NA	NA
027161	18	M	NA	NA

Ten (10) pediatric ESFT patients and 6 non-oncology pediatric patients were utilized for the analysis Ewing Sarcoma derived small extracellular vesicles (sEV).

## DISCUSSION

As with the majority of pediatric malignancies, translational research in ESFT lags behind work being done in adult oncology. In this study, we have sought to advance the scientific literature of ESFT by defining for the first time the proteome of ESFT-associated sEVs. The idea of a “liquid biopsy” has prompted a plethora of studies on sEV biomarkers in cancer [45]. However, isolation of tumor-associated sEVs directly from patient blood samples is particularly challenging, in part, due to lack of specific markers capable of distinguishing cancer from non-cancerous derived sEVs. We hypothesized that the we could utilize protein biomarkers towards the enrichment of ESFT-associated sEVs, given the *EWS-ETS* fusion transcript is not readily detected in plasma. Additionally, we speculated that, in doing this, we could significantly increase the sensitivity in detection of otherwise low frequency mutations, *i.e., EWS-ETS*, which is diagnostic of the disease. By initiating studies to develop a liquid-based biopsy we sought to improve the molecular tools for the diagnosis, detection, and disease monitoring of patients with ESFT.

The use of blood-based diagnostics, referred to as liquid biopsies, provides an opportunity improve diagnosis and to monitor disease states in real-time [46]. Circulating tumor cells (CTCs) and circulating tumor DNA (ctDNA) have been used clinically, but their diagnostic value is still limited for many cancer types based on sensitivity and specificity of the respective assays. For example, the heterogeneity and rarity of CTCs define the challenges of purifying an extremely small number of CTCs from a large number amount of other cells in a large blood volume (typically 7.5 mL) [47]. In metastatic cancer setting, CTCs within the peripheral circulation occur at an estimated number of one CTC per 1 × 10^5–7^ peripheral blood mononuclear cells [48]. Relevant to our studies, Benini *et al.* reported that CD99^+^ CTCs were detected in 4 of 23 peripheral ESFT using 10-mL blood samples from patients age range of 13–32 years. ctDNA is another option being promoted as an alternative noninvasive method that overcome many difficulties related to tissue biopsy (*e.g.,* spectrum of mutations limited to a single region of the tumor, serial sampling usually not feasible, etc.). Though significant progress has been accomplished in the field of ctDNA diagnostics, especially those based on next generation sequencing, serious limitation exists, given the vast majority of circulating DNA is primarily composed of normal cell free DNA (cfDNA) [49, 50]. Relevant to our studies, Shulman *et al.* utilizing an NGS hybrid capture assay and an ultra-low-pass whole-genome sequencing assay to detect ctDNA in a median of 2 mL of banked localized ESFT pediatric patient plasma from Children’s Oncology Group (COG), demonstrated ctDNA in 53.3% (41/77) of newly diagnosed patients [51]. Allegretti *et al.* demonstrated that *EWS-FLI1* Type I and Type II rearrangements could be identified, regardless of patient-specific *EWS-FLI1* DNA breakpoints in circulating tumor RNAs (ctRNAs) in 4 patients (1 metastatic and 3 localized) ranging in ages from 8–45 years utilizing 1.8 mLs of plasma. Although the most frequent translocation partner of *EWS* is *FLI1*, with the common fusion joining *EWS* exon 7 in frame with *FLI1* exon 6 (type 1 fusion), there are several other *EWS*-*FLI1* type fusions, as well 5–10% of patients with ESFT have an EWS partner *ERG*. Our method focused on ESFT exo-proteins to immuno-enrich tumor-associated circulating EVs for the subsequent detection of *EWS-FLI1* Types I, II, and III and *EWS-ERG* fusion transcripts within pediatric patient plasma. ESFT is regarded as a malignancy of childhood and adolescence and thus rare in over the age of 40, hence our focus on enrolling patients who consist of the majority of this disease population in this assay. A limitation in pediatric studies such as this is in part due to the incidence of ESFT in children and young adults within the United States and the volume of blood ethically and safely obtainable, hence we have utilized 10 pediatric patients in this study and were able to detect *EWS-ETS* fusion transcripts in both metastatic and localized subset of patients utilizing only 250 μL of plasma. We have expanded and improved upon prior published accomplishments [52–54] by directly identifying ESFT sEV-associated protein biomarkers which enabled us to enrich for ESFT-specific sEVs (AUC = 0.92) and concordantly detecting the *EWS-ETS* fusion transcript (PPV = 1.00 and NPV = 0.67) from as little as 250 μl of archival plasma samples. The approach by Benini *et al.* [55], Allegretti *et al.* [56], and our own circumvent the requirement to sequence patient-specific breakpoints, obtain long tumor DNA fragments from fresh tumor, and design patient-specific primer sets. Based on the above technical background, our clinically relevant assay could be applied to diagnose and potentially monitor ESFT patients during therapy and then off therapy for recurrence of disease.

In this first proteomic analysis of ESFT derived sEVs, we demonstrate the presence of 618 core enriched ESFT-sEV proteins, including ESFT associated proteins such as CD99/MIC2, caveolin, and GLG1 which have recently been proposed as markers for ESFT [57]. Among the top sEV biomarker candidates, we identified both HINT1 and NGFR (p75NTR). Previously, HINT1 was found to repress β-catenin-mediated transcription of Wnt target genes, and had been noted to be differentially expressed between localized and metastatic ESFT [44]. NGFR, also known as low-affinity nerve growth factor, a member of the tumor necrosis receptor family and has been implicated in the paracrine growth regulation of a number of neuronal as well as non-neuronal tumor types [58], such as prostate cancer, invasive ductal breast cancer, pancreatic carcinoma and malignant melanoma. NGFR is abundantly expressed during development but in adult organisms is known to be downregulated. However, the NGFR is re-expressed in conditions of increased neuronal cell death [59]. In a study done by Fanburg-Smith and Miettinen, non-neural mesenchymal tumors showed variable NGFR expression based on tumor type, with rhabdomyosarcoma demonstrating a 90% positivity of 94 cases, Ewing Sarcoma 32% in 31 cases and extraskeletal osteosarcomas 23% in 13 cases [60]. Likewise, we observed over 80% of tissue samples positive for NGFR by IHC. Both CD99 and NGFR on subsequent analyses of the mass spectrometry sEV proteomics utilizing Proteome Discoverer v2.3.0.523 confirmed that both of these proteins are in the top 15% based on the respective MS1 data and PSM. In addition to ESFT-specific proteins, the detection of chimeric mRNAs transcribed from the *EWS-ETS* fusion genes are a valuable tool in the molecular diagnosis of ESFTs [61]. Overall the ESFT genome is genetically quiet with few genomic aberrations/mutations identified compared with most cancers [62–64]. In recent years, it has become abundantly clear that the *EWS-ETS* rearrangements are the most important molecular determinant of tumorigenesis and progression of the ESFT [65]. This makes the identification of ESFT related proteomics and translocations in circulation particularly appealing because they are not likely to be lost during tumorigenesis and tumor progression. This current study is the first to identify the *EWS-ETS* transcript with high specificity in sEVs isolated from pediatric ESFT patient plasma samples by combining sEV proteomics and immuno-enrichment techniques.

The composition of sEVs is not sporadic in nature, suggestive that the incorporation of proteomic and RNA cargo into sEVs are a regulated process. Different forms of post translational modifications of proteins have been reported to occur in sEVs, such modifications permit for protein versatility via influencing their activation state, subcellular localization, stability, and protein: protein interactions [66]. Certain aspects of post-translational modification have been shown to be integrated within the biopathway of sEV release. The generation and progression of many diseases have been associated with sEV-mediated transport of misfolded disease-causing proteins as well [67]. Loading of proteins within sEVs because of protein damaging modifications such as oxidation [68] or misfolded proteins has also been well described [69]. Our results suggest that some proteins within ESFT derived-sEVs/exosomes, including EWS-ETS fusion proteins, are likely post-translationally modified prior to sorting. The presence of abundantly enriched proteins on the membrane of sEVs as well as those of modified proteins in sEVs offers an excellent opportunity to develop highly specific techniques for the isolation and identification of sEVs for biomarker utility; thus, offering an unprecedented opportunity to garner information for pediatric sarcomas in a non-invasive manner and help potentially design curative options that would further improve on the OS of these patients. A crucial challenge to our assay is in achieving absolute sensitivity, while avoiding any false positives. The latter is not a major issue given that *EWS-ETS* fusion are uncommon in other cancer types; however, the presence of the *EWS-ETS* transcript is as low as < 1 copy/10^5^ sEVs as we have previously published [52], which is a likely reason for our inability to achieve 100% specificity with the current assay. There are several approaches that may further enhance aspects of our technique. Effective combinations of antibodies towards ESFT sEVs membrane-based antigens discovered through our proteomics alongside NGFR and CD99 for immunocapture, rather than dual-antibody approaches implemented within our study, may potentially further improve sEV isolation. Another approach would be to increase the volume of plasma input into our assay to 500–1,000 μL. Prior studies have demonstrated approximately 2.11% of the total RNA content within sEVs are mRNA fragments, while microRNAs are vastly enriched within these circulating extracellular vesicles. We are currently studying the utility of ESFT sEV miRNAs as biomarkers in conjunction with our *EWS-ETS* detection methodologies. By incorporating several markers for the detection and diagnosis of ESFT, a biomarker signature as such will further increase the sensitivity of this assay to enable the identification of even minimal residual disease presence during therapy and even post-therapy.

In order to further advance the development of sEVs as biomarkers in ESFT, our ongoing studies are integrating protein markers identified through our study into our prototypic microfluidic chip. As discussed above, we have recently demonstrated the quantitative measurement of *EWS-FLI1* mRNA copy numbers in pPNET-derived sEVs [52]. Although a rare disease, development of this type of integrated assay could aid in the diagnosis of all members of the ESFT family. We are currently in process of developing of single microfluidic platform using our validated capture reagents to streamline the enrichment of tumor derived sEVs and quantitative measurement of *EWS-ETS* fusion transcripts in ESFT. Studies of ESFT tumor derived sEVs may reveal possible new important therapeutic targets, as well as perhaps yield RNAs prognostic for tumor aggressiveness and chemotherapeutic sensitivity allowing clinicians to better treat this pediatric malignancy.

## MATERIALS AND METHODS

### Cell line and culture conditions

Hs919. T, SK-ES-1, RD-ES were purchased from the American Type Culture Collection. In addition, TC-71, COG-E-352, and CHLA-258 cell lines were obtained from the Children’s Oncology Group (COG). All cell line identities were confirmed by short tandem repeat profiling via the Clinical Molecular Oncology Laboratory at KUMC and the causative mutations in ESFT were confirmed by FISH utilizing the *EWSR1* break apart probes for EWS translocation at Children’s Mercy Clinical Genetics and Genomics Laboratories. All cell lines were cultured at 37°C under a 5% humidified CO_2_ atmosphere. TC-71, COG-E-352 and CHLA-258 cell lines were maintained in Iscoves Modified Dulbecco’s Medium (IMDM), supplemented with L-glutamine (3 mM), insulin, and transferrin (5 mg/ml each), selenium (5 ng/ml), and 20% heat-inactivated exosome free FBS (whole medium). RD-ES cell line was maintained in RPMI 1640 with L-glutamine, supplemented with 15% heat-inactivated exosome free FBS (whole medium). SK-ES-1 was maintained in McCoy’s 5A with L-glutamine supplemented with 15% heat-inactivated exosome free FBS (whole medium). Hs919. T cells were maintained in DMEM with high glucose with L-glutamine, supplemented with 20% heat-inactivated exosome free FBS (whole medium). All cell lines were cultured in the presence of 10% penicillin streptomycin to prevent bacterial growth/contamination.

### sEV isolation from conditioned medium of cultured cells

ESFT cell lines were grown and cultured in five T175 cm^2^ flasks containing 10% exosome-free FBS medium for 48–72 hours until cellular sub-confluency of ~70% was reached. The *benign* osteoid osteoma control cell line, Hs919. T was cultured in five T175 cm^2^ flasks in 20% exosome-free FBS media for 168–240 hours until cellular sub-confluency of ~60–70%. Media were collected and immediately centrifuged at 2,500 rpms for 5 minutes to eliminate cellular debris. A total of 150 mL of conditioned medium was collected and ultra-centrifuged at 4°C for 45 minutes at 8,700 rpm (10,000 × *g*). The supernatant was then collected and ultracentrifuged again at 4°C for 75 minutes at 28,800 rpms (110,000 × *g*). sEV pellets were washed with PBS and were collected by ultracentrifugation at 4°C for 60 minutes at 35,800 rpms in Beckman Coulter Quik-Seal Centrifuge Tubes. Finally, each sEV pellet was resuspended in 50–100 μl of PBS based on pellet size and then stored at –80°C.

### Nanoparticle tracking analysis (NTA)

Size and concentration of isolated and purified cell line derived sEV analysis was via the NanoSight LM10 (NanoSight Ltd., Minton Park, Amesbury, UK). A 1:1500 sEV pellet dilution in PBS was used for this analysis. NTA is a system for particle size analysis ranging from 30–1,000 nm, with lower detectable limits dependent on the refractive index of the nanoparticles. This technique combines laser light scattering microscopy with a charge-coupled device (CCD) camera, enabling the visualization and recording of nanoparticles within solution.

### sEV proteomics analysis

ESFT cell line derived sEV were reduced with 0.1 M DTT for 60°C for 30 min prior to dilution into Filter-Aided Sample Preparation (FASP) buffer (8 M urea, 0.1 M Tris-HCl pH 8.5) and transferred to a Microcon-10 (EMD Millipore, Billerica, MA, USA) 10kDa centrifugal for trypsinization by the FASP method [70, 71]. Tryptic digests were trap cleaned using C18 PROTO^™^ Ultra MicroSpin columns (Nest Group, Inc, Southborough, MA) then lyophilized and redissolved into 2% acetonitrile/0.1% formic acid prior to LCMS analysis. Tryptic peptides were separated using an EASY n-LC (Thermo) UHPLC system and a 360 μm OD × 100 μm ID fused silica tip packed with 10 cm of Jupiter 5 μm C18 300 Å material (Phenomenex, Torrance, CA, USA). Following injection of the sample onto the column, separation was accomplished with a 75 min linear gradient from 2% acetonitrile to 40% acetonitrile in 0.1% formic acid. The eluate was introduced into the LTQ-Velos-Orbitrap ELITE + ETD mass spectrometer using a Nanospray Flex source (ThermoElectron, Waltham, MA, USA). An Orbitrap Elite – ETD mass spectrometer (ThermoElectron) was used to collect data from the LC eluate. An Nth Order Double Play with ETD Decision Tree method was created in Xcalibur v2.2. Scan event one obtained an FTMS MS1 scan (normal mass range; 60,000 resolution, full scan type, positive polarity, profile data type) for the range 300–2000 m/z. Scan event two obtained ITMS MS2 scans (normal mass range, rapid scan rate, centroid data type) on up to ten peaks that had a minimum signal threshold of 20,000 counts from scan event one. Each sample was injected twice yielding essentially a technical replicate, to aid with observation and ID of low abundant proteins. A decision tree was used to determine whether CID or ETD activation was used. An ETD scan was triggered if any of the following held: an ion had charge state 3 and m/z less than 650, an ion had charge state 4 and m/z less than 900, an ion had charge state 5 and m/z less than 950, or an ion had charge state greater than 5; a CID scan was triggered in all other cases. The lock mass option was enabled (0% lock mass abundance) using the 371.101236 m/z polysiloxane peak as an internal calibrant. Initially data dependent spectra search was directed by Proteome Discoverer v1.3.0.330 (ThermoElectron) using Mascot v2.1 and Sage-N Sorcerer Sequest algorithms and the UniprotKB Homo sapiens reference proteome canonical and isoform sequences (7/10/2013 version). Search parameters included: variable methionine oxidation, fixed cysteine carbamidomethylation, up to 2 missed tryptic cleavages, 50 ppm precursor error for MS1 Orbitrap FTMS data, 0.8 Da error for CID-based MS2 LTQ data and 1.2Da error for ETD-based MS2 data. In order to estimate the false discovery rate, a decoy database was generated from this database with the program decoy. pl (from http://www.matrixscience.com/).

The ESFT cell line derived sEV mass spectrometry proteomics data was evaluated by Proteome Discoverer v2.3.0.523 (ThermoElectron) for imputation, match-between-runs, normalization steps, and protein modification for carbamylation to address effects of urea introduced during the FASP protocol. Search parameters included: variable methionine oxidation, fixed cysteine carbamidomethylation, up to 2 missed tryptic cleavages, 50 ppm precursor error for MS1 Orbitrap FTMS data, 0.8 Da error for CID-based MS2 LTQ data and 1.2Da error for ETD-based MS2 data. Technical duplicate data were searched and integrated as one sample. In order to estimate the false discovery rate, a decoy database was generated from this database with the program decoy. pl (from http://www.matrixscience.com/). For samples with peptides not confidently identified via Proteome Discoverer v2.3.0.523 utilized mass tolerance windows and chromatographic alignments to determine peptide presence. With presence of an m/z signal, area of the ion was extracted and utilized to populate missing values. For runs without m/z values within the mass accuracy window nor chromatographic retention time then no value was recorded. MS1 area values for peptides with high confident MS2 data were represented as high signal levels (green values). MS1 area values for peptide features that match within mass accuracy tolerance and retention time tolerance to high confidence data in a separate LCMS run were represented as peak found but not sufficient for MS2 validation (yellow values). For non-detectable proteins without sufficient information to assign high confidence spectra (green value) nor with mass accuracy or retention time tolerances (yellow data), no signal or red value was assigned.

### Bioinformatics analysis

Initial analysis of mass spectrometry proteomics dependent spectra directed by Proteome Discoverer v1.3.0.330 identified 1082 proteins. Protein identity and quantitative data were exported for further statistical and bioinformatic analysis. The list of differentially enriched proteins in sEV were submitted to identify the enrichment of biological processes in sEV according their gene ontology annotation extracted from the UniProt database. Qualitative data generated from proteomic mass spectrometric analysis were analyzed using Ingenuity Pathway Analysis (IPA; Ingenuity System, Redwood city, CA, USA; http://www.ingenuity.com), Plasma Proteome Database (http://www.plasmaproteomedatabase.org/) and Exocarta Vesiculopedia to identify potential biomarker sEV signatures and their application in pediatric sarcomas.

Quantitative data from proteomic mass spectrometric analysis were analyzed using the R statistical programming language (http://cran.r-project.org) using R version 3.6.1. For each biological replicate, there were two technical replicates in which total spectral counts were available. If the total spectral count was “0” or “NA” it was replaced with a value of “0.5”. These total spectrum counts were averaged for each pair of technical replicates and then log2 transformed for analysis. Protein expression data collected from ESFT cell line derived sEVs from EWS-FLI1 Type I fusion (TC-71, *n* = 2), EWS-FLI1 Type II fusion (SK-ES-1, *n* = 1 and RD-ES, *n* = 1), EWS-FLI1 Type III fusion (CHLA-258, *n* = 2), and EWS-ERG fusion (COG-E-352, *n* = 1) was log_2_ transformed prior to analysis. Due to the lack of biological replicates for some of the cell lines, one-way analysis of variance (ANOVA) models were fit independently to each of the proteins and linear contrasts were used to test the comparisons of interest. *P*-values and 95% confidence intervals were calculated for each of the linear contrasts. Volcano plots were generated for each comparison of interest with the difference in mean log_2_ (expression) plotted versus the -log_10_ (*p*-value).

To identify disrupted biological pathways between EWS-FLI1 versus EWS-ERG cell lines, a gene set enrichment analysis (GSEA) was implemented in R using the package gage [72]. Protein expression data (total spectrum count) from the EWS-FLI1 (*n* = 6) and EWS-ERG (*n* = 1) cell lines were used as the basis of comparison. The predefined gene set used was derived from KEGG pathways and stored in the 'kegg.gs' data set in the gage package.

### SDS-PAGE and western blot analysis

sEV samples and cell lysates were prepared in radioimmunoprecipitation assay (RIPA) buffer and separated by adding 40 μg protein on 7%, 10%, or 4–20% Mini-PROTEAN^®^ TGX™ Precast Gels, (BioRad, Hercules, CA, USA). The completed gels were transferred to a supported nitrocellulose membrane (BioRad). The membranes were blocked with 5% non-fat milk for one hour with gentle rocking. Membranes were incubated with primary antibodies overnight and then washed thrice for 10 minutes before addition of HRP conjugated anti-rabbit or anti-mouse secondary antibody (BioRad) for 1 hour. Membranes were subsequently washed and treated with ECL Western Blotting Substrate (Fisher Scientific) according to manufacturer’s instructions and antibodies used were either rabbit or mouse Isotype (Supplementary Table 8).

### Immuno-pulldown and analysis of specific population of plasma-derived sEVs

Immuno-pulldown of sEVs required two overnight 18 to 20-hour incubation steps. Day 1 consisted of Dynabead™ washes and immobilization of antibodies. Dynabead Wash-1,000 μg (100 μL) of Dynabeads™ M-270 Streptavidin beads were washed three times in PBS Buffer. Immobilization of Antibodies-10 μg of biotinylated antibody were incubated with beads overnight with 1,000 μg of Dynabeads. Day 2 consisted of Dynabead washes and immobilization of exosomal proteins. Dynabead Wash-antibody coated beads were separated by use of a magnet. Supernatants were removed and subsequently beads were washed 4–5 times with PBS containing 0.1% BSA. Antibody coated beads were then resuspended in 100 μL. A total of 50 μL of the antibody coated bead suspension was placed in 250 μL of plasma and incubated overnight to immobilize sEV membranous proteins. Day 3 consisted antibody coated beads exosomal conjugate washes with PBS and then separation with Exoquick™ Exosome Precipitation solution (System Biosciences) with magnet to dissociate the biotin-streptavidin bond.

### qRT-PCR of exoEWS-ETS fusion transcripts

RNA isolated from cells and sEV samples were by utilizing the miRNeasy kit (Qiagen) in combination with phase lock tubes (5-Prime) according to the manufacturer protocols for total RNA isolation. Prior to RNA isolation, sEV pellets were treated with RNAse A (Thermo Fisher) at a concentration of 5 μg/ml for 30 minutes at 4°C. cDNA was made from 50–100 ng total RNA and using SuperScript IV VILO cDNA synthesis kit (Thermo Fisher). cDNA from cell lines was diluted (1/4) with TE buffer. cDNA from sEV samples was not diluted further. For detection of the EWS-FLI1 types I, II, and III transcript we used the forward primer EWSF 5′-GCCAAGCTCCAAGTCAATATATAGCCAACAG-3′, and the reverse primer FLIR 5′-GGGCCGTTGCTCTGTATTCTTACTG-3′. For the detection of *EWS-Erg* fusion transcript we utilized the EWSF forward primer with the reverse primer ERGR1 5′-GAGTTGGAGCTGTCCGACAGG-3′. For each transcript we used a Fam-labeled probe 5′-GCAGCAGCTACGGGCAGCAG-3′. All primers and probes were manufactured by IDT. qRT-PCR assays were run on a CFX96 (BioRad) in volumes of 20 μl according to the following conditions: 50°C, 3 minutes; 95°C, 10 minutes; then 40 cycles of 95°C, 15 seconds; and 54°C, 60 seconds.

### Pediatric sarcoma patient biobanking

The translational aspect of this study was conducted within the framework of a newly initiated Children’s Mercy Kansas City IRB approved protocol (PI: G. Samuel, CMH IRB# 13010015), which is an ongoing, prospective observational single-center study. Enrollment criteria were for patients less than 18 years of age with newly diagnosed pediatric sarcomas focused primarily on pediatric ESFT, Rhabdomyosarcoma and Osteosarcoma. Patients were recruited while either on the inpatient or outpatient setting of Children’s Mercy Kansas City Department of Hematology Oncology and Bone Marrow Transplantation starting December 2012; recruitment is ongoing. All newly diagnosed and recurrent patients were enrolled; a few patients actively undergoing therapy were also enrolled at the time of initiation of this protocol. The majority of enrolled patients had not received chemotherapy or radiotherapy prior to study inclusion. Parents of eligible patients aged below 7 years gave written informed consent. Patients 7 years and above were also asked to provide written assent. Samples were collected prior to initiation of therapy, immediately prior to each cycle of therapy and then every three months off therapy for the first two years only. At enrollment, data on medical history were collected including demographic and clinical details of tumor site, histology and stage were documented. During the following observation period further blood samples were taken prior to each cycle of chemotherapy or prior to local control (surgery and/or radiation therapy).

### Statistical analysis

All statistical analyses were performed using R (http://cran.r-project.org). A one-way analysis of variance model (ANOVA) was used to compare the number of EVs (particles per mL) assessed in plasma samples collected from healthy control individuals (*n* = 6) and clinical pediatric ESFT patients (*n* = 10). Briefly, the number of EVs was modeled as the dependent variable, against a single independent variable representing the health status of the individual (*e.g.,* healthy plasma, ESFT localized, and ESFT metastatic). Parameter estimates obtained from the one-way ANOVA model were used to construct specific contrasts, which included a comparison of the mean number of EVs between: ESFT (both localized and metastatic) versus healthy, localized ESFT versus healthy, metastatic ESFT versus healthy, and localized ESFT versus metastatic ESFT. The positive predictive value (PPV) and negative predictive value (NPV) for detecting the EWS-ETS fusion transcript were computed using *epitests* function in the R package epiR. Confidence intervals for the PPV and NPV were calculated using a previously described procedure [70].

## SUPPLEMENTARY MATERIALS











## Abbreviations

ESFT: Ewing Sarcoma Family of Tumors
sEVs: small Extracellular Vesicles
PNET: peripheral primitive neuroectodermal tumors
MVB: multivesicular bodies
EFS: Event Free Survival
OS: Overall survival
CTCs: Circulating tumor cells
ctDNA: circulating tumor DNA
