# Supplementary material for: Ewing sarcoma family of tumors-derived small extracellular vesicle proteomics identify potential clinical biomarkers
Source: Oncotarget. 2020 Aug 4;11(31):2995–3012. doi: 10.18632/oncotarget.27678 (PMC7415402; doi:10.18632/oncotarget.27678)
Supplement: Supplementary file 1 [file oncotarget-11-2995-s001.pdf]

Ewing sarcoma family of tumors-derived small extracellular vesicle proteomics identify potential clinical biomarkers

SUPPLEMENTARY MATERIALS

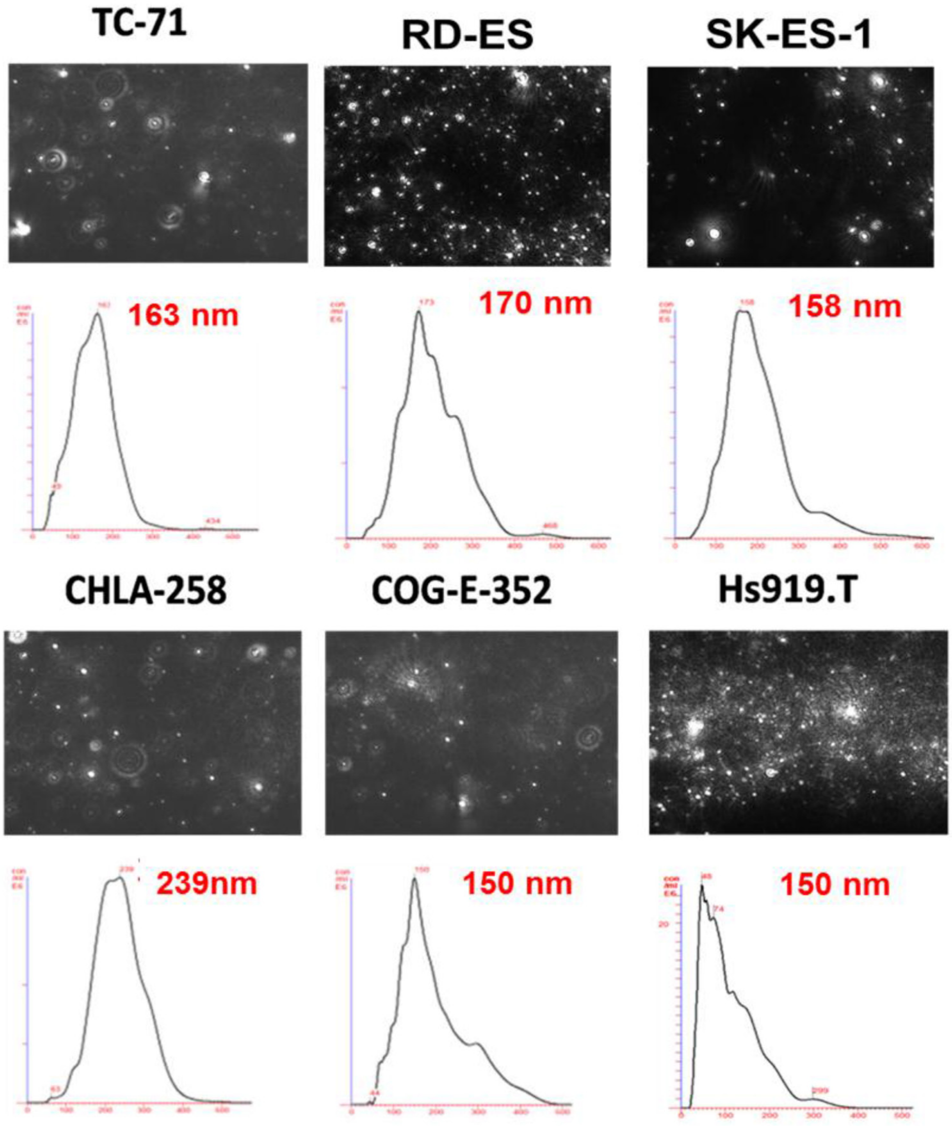

Supplementary Figure 1: Size distribution analysis of microvesicles. The size and number of microvesicles isolated from ESFT cell lines as determined by NTA analysis.

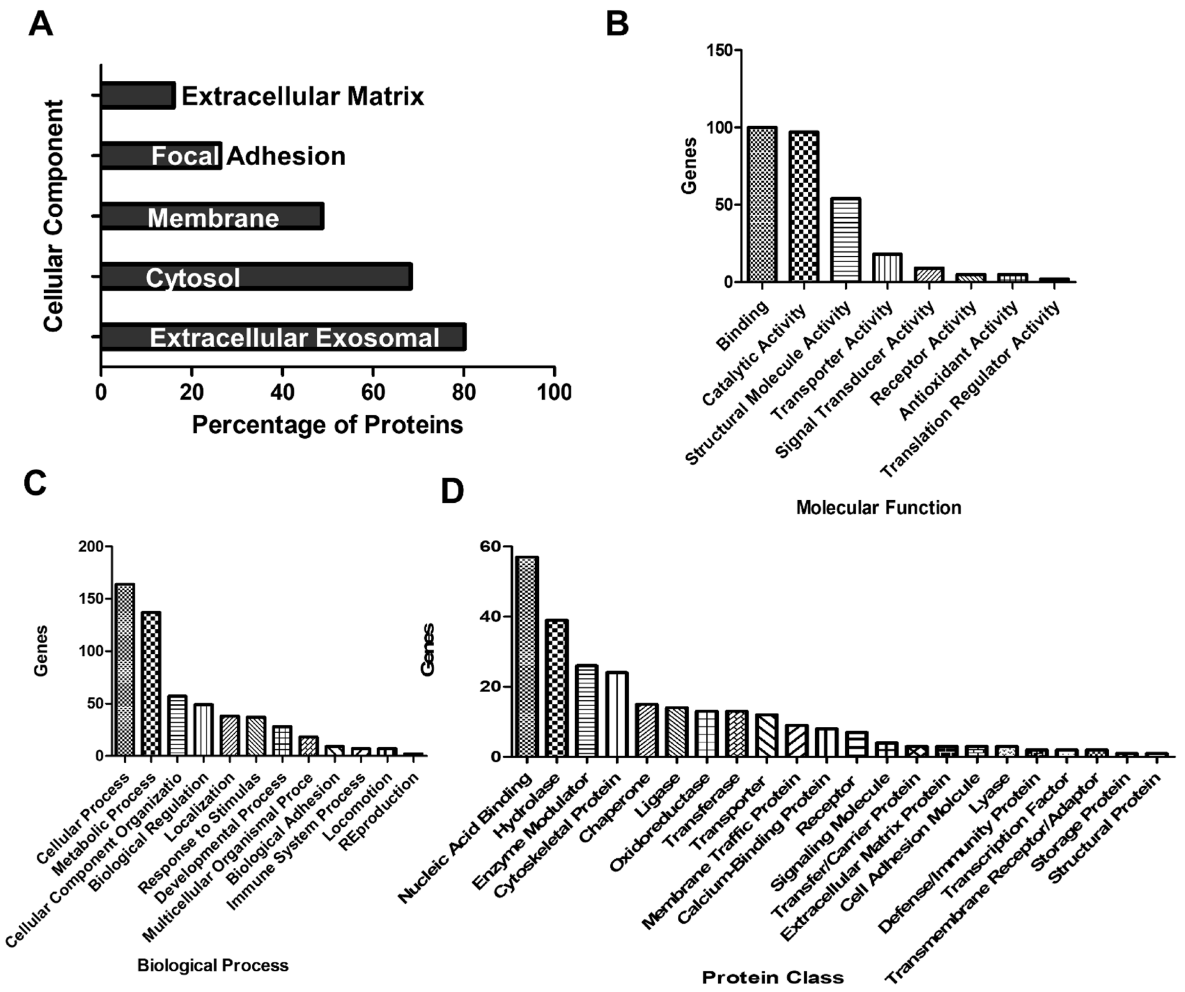

Supplementary Figure 2: Pathway analysis of ESFT sEV core proteome. (**A**) FunRich analysis of common ESFT sEV proteins. (**B**–**D**) PANTHER (Protein Analysis Through Evolutionary Relationships) analysis results of molecular functions (B), biological functions (C), and protein classes of ESFT sEVs (D).

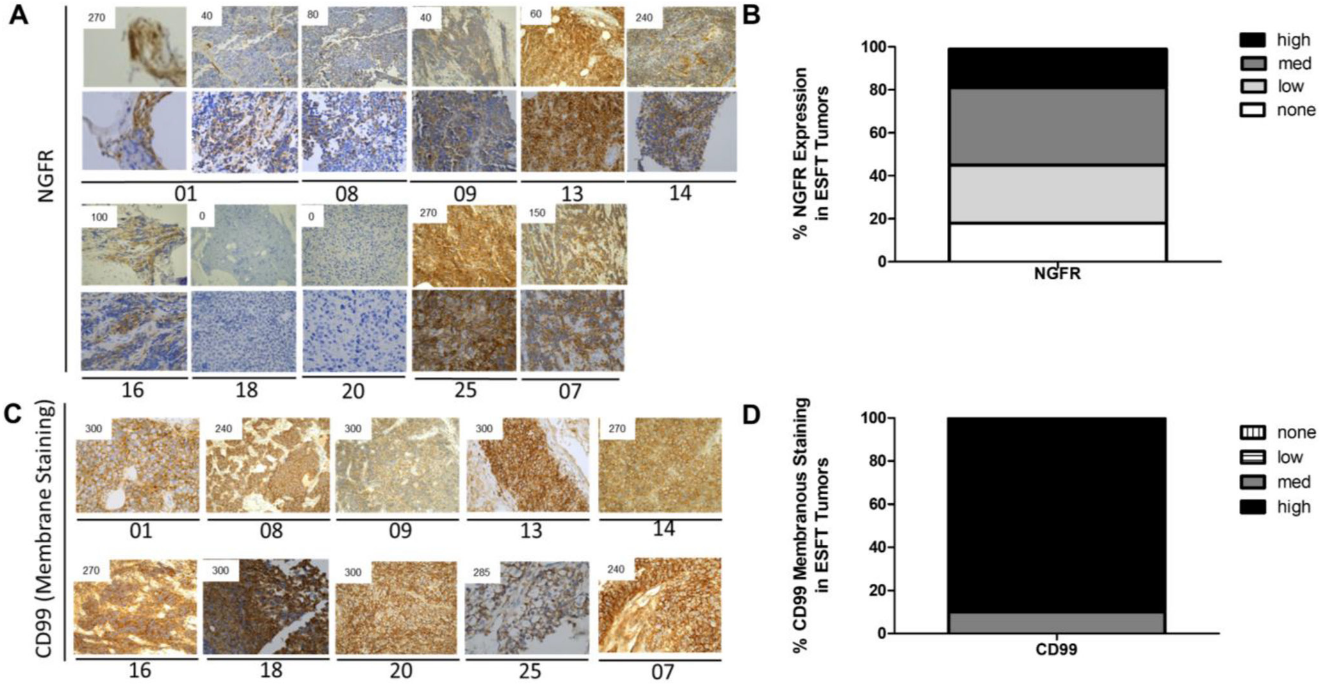

Supplementary Figure 3: IHC analysis of CD99 and HINT1 in ESFT tissue samples. (**A**–**B**) NGFR staining. (**C**–**D**) CD99 staining.

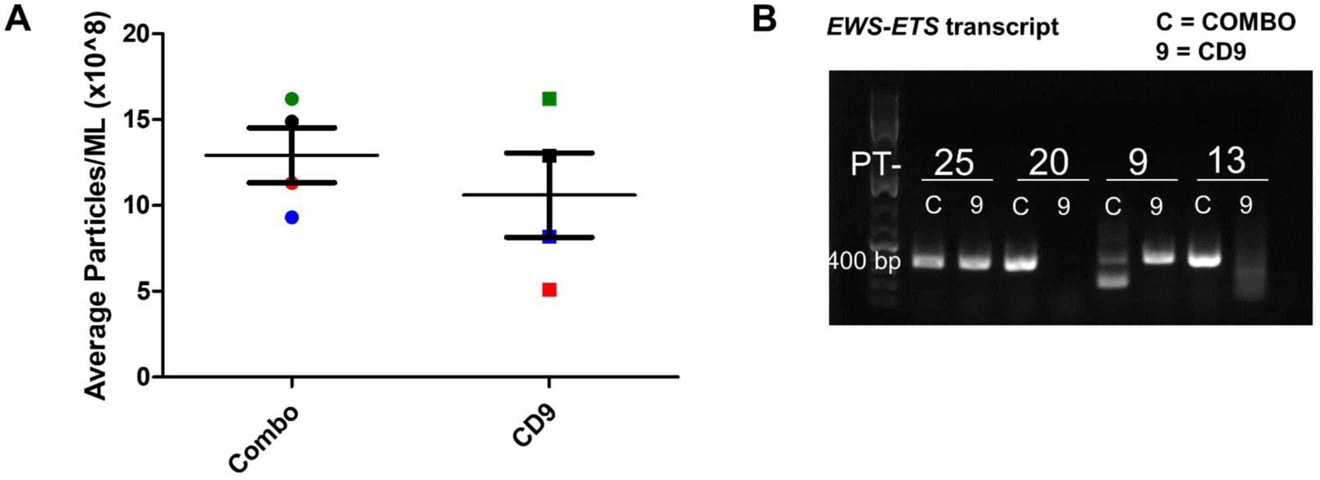

Supplementary Figure 4: Comparison of ESFT sEVs isolated through COMBO or CD9 immuno-isolation in 250 µL of pediatric clinical plasma samples. (**A**) The average number of sEVs isolated demonstrated no significant variation depending on technique. (**B**) DNA gel electrophoresis of EWS-ETS amplicon (~400 bp) illustrates the presence of the transcript in all of the patient samples *vs.* 50% of the samples isolated using the common EV tetraspanin CD9 alone.

Supplementary Table 1: Cell line information

Cell Line	Age (years)	Diagnosis	t(11;22)	Fusion Type	Phase of Therapy	Clinical Status
TC-71	22	Ewing Sarcoma	+	Type I	Post-therapy	Progressive Disease
RD-ES	19	Ewing Sarcoma	+	Type II	-	-
SK-ES-1	18	Ewing Sarcoma	+	Type II	-	-
CHLA-258	14	PNET	+	Type III	Post-therapy	Progressive Disease
COG-E-352	17	PNET	-	EWS-ERG	Post-therapy	Recurrence
Hs919.T	34	Benign Osteoid Osteoma	-	None	-	-

Data related to 5 ESFT cell lines comprised of both EWS-FLI1 and EWS-ERG oncogenic fusions and Hs919.T a benign bone tumor control cell line utilized in this study.

Supplementary Table 2: ESFT cell line derived sEV proteomic data. See Supplementary Table 2

Supplementary Table 3: Analysis of ESFT cell line derived sEV proteomic data. See Supplementary Table 3

Supplementary Table 4: Differential protein expression comparisons in ESFT sEVs. See Supplementary Table 4

Supplementary Table 5: Gene Set Enrichment Analysis (GSEA) comparison of EWS-FLI1 sEV and EWS-ERG sEV proteome. See Supplementary Table 5

Supplementary Table 6: Proteins enriched in ESFT sEV sample

Symbol	Name	Symbol	Name
ACTG1	actin gamma 1	HSPA5	heat shock 70kDa protein 5 (glucose-regulated protein, 78kDa)
ACTR3	ARP3 actin-related protein 3 homolog (yeast)	HSPA8	heat shock 70kDa protein 8
ALDOA	aldolase A, fructose-bisphosphate	HSPD1	heat shock 60kDa protein 1 (chaperonin)
ALDOC	aldolase C, fructose-bisphosphate	HUWE1	HECT, UBA and WWE domain containing 1, E3 ubiquitin protein ligase
ANXA1	annexin A1	ITGA5	integrin, alpha 5 (fibronectin receptor, alpha polypeptide)
ANXA2	annexin A2	ITGB1	integrin, beta 1 (fibronectin receptor, beta polypeptide, antigen CD29 includes MDF2, MSK12)
ANXA5	annexin A5	JAK1	Janus kinase 1
ARHGDIA	Rho GDP dissociation inhibitor (GDI) alpha	KRT1	keratin 1, type II
BSG	basigin (Ok blood group)	KRT14	keratin 14, type I
CALR	calreticulin	KRT9	keratin 9, type I
CAV1	caveolin 1, caveolae protein, 22kDa	MYH9	myosin, heavy chain 9, non-muscle
CD99	CD99 molecule	NGFR	nerve growth factor receptor
CTNNA1	catenin (cadherin-associated protein), alpha 1, 102kDa	NPM1	nucleophosmin (nucleolar phosphoprotein B23, numatrin)
CTNNB1	catenin (cadherin-associated protein), beta 1, 88kDa	PDIA3	protein disulfide isomerase family A, member 3
EIF2S1	eukaryotic translation initiation factor 2, subunit 1 alpha, 35kDa	PFN1	profilin 1
ENO1	enolase 1, (alpha)	PHB	prohibitin
ENO2	enolase 2 (gamma, neuronal)	PKM	pyruvate kinase, muscle
EZR	ezrin	PLG	plasminogen
F2	coagulation factor II (thrombin)	PRDX1	peroxiredoxin 1
FASN	fatty acid synthase	SLC16A1	solute carrier family 16 (monocarboxylate transporter), member 1
FLNA	filamin A, alpha	SLC7A5	solute carrier family 7 (amino acid transporter light chain, L system), member 5
FLNC	filamin C, gamma	STMN1	stathmin 1
FSCN1	fascin actin-bundling protein 1	TFRC	transferrin receptor
FTH1	ferritin, heavy polypeptide 1	THBS1	thrombospondin 1
GAPDH	glyceraldehyde-3-phosphate dehydrogenase	TLN1	talin 1
GSTP1	glutathione S-transferase pi 1	TPI1	triosephosphate isomerase 1
H2AFX	H2A histone family, member X	TPM3	tropomyosin 3
HINT1	histidine triad nucleotide binding protein 1	UCHL1	ubiquitin carboxyl-terminal esterase L1 (ubiquitin thiolesterase)
HLA-A	major histocompatibility complex, class I, A	VCL	vinculin
HNRNPH1	heterogeneous nuclear ribonucleoprotein H1 (H)	XRCC5	X-ray repair complementing defective repair in Chinese hamster cells 5 (double-strand-break rejoining)
HSPA4	heat shock 70kDa protein 4	YWHAG	tyrosine 3-monooxygenase/tryptophan 5-monooxygenase activation protein, gamma

A total of 62 potential ESFT biomarker candidates were identified, which included both CD99/MIC2 and HINT1 in the ESFT sEV proteome.

Supplementary Table 7: ESFT sEV proteins with biomarker applications

Protein Symbol	Gene	Location	Application
HINT1	Histidine triad nucleotide binding protein 1	Intracellular	Prognosis
CAV1	Caveolin 1	Plasma Membrane	Diagnosis
NPM1	Nucleolar phosphoprotein B23	Intracellular	Disease Progression
CTNNB1	Cadherin-associated protein beta 1	Intracellular	Diagnosis<br>Disease Progression<br>Prognosis
ENO2	Enolase 2	Intracellular	Diagnosis<br>Prognosis
EZR	Ezrin	Plasma Membrane	Prognosis
ITGA5	Integrin, alpha 5	Plasma Membrane	Prognosis
JAK1	Janus kinase 1	Intracellular	Efficacy
CD99	CD99 molecule	Plasma Membrane	Diagnosis
NGFR	Nerve growth factor receptor	Plasma Membrane	Disease Progression<br>Prognosis

A total of 10 proteins previously identified to be associated with sarcomas were further investigated. 4 of the 10 proteins had been previously identified as diagnostic biomarker potential in sarcomas, while 6 of the 10 proteins were identified as prognostic biomarker potential in sarcomas.

Supplementary Table 8: Antibodies

Antibodies	Isotype	Source
CD99 (1C3)	Rabbit	Thermo Fisher
NGFR (D4B3)	Rabbit	Cell Signaling
HINT1	Rabbit	ORIGENE
ITGA5	Rabbit	Cell Signaling
JAK1	Rabbit	Thermo Scientific
CTNNB1 (14/Beta-Catenin)	Mouse	BD Biosciences
EZR (3C12)	Mouse	Sigma Aldrich
ENO	Rabbit	Cell Signaling
NPM1 (NA24)	Mouse	Santa Cruz
β-actin (AC-15)	Mouse	Sigma Aldrich

10 antibodies utilized for immunoblot analysis in ESFT sEV samples.
